# *Notes from the Field*: Errors in Administration of an Excess Dosage of Yellow Fever Vaccine — United States, 2017

**DOI:** 10.15585/mmwr.mm6703a6

**Published:** 2018-01-26

**Authors:** Michael M. McNeil, Beth F. Hibbs, Elaine R. Miller, Maria V. Cano

**Affiliations:** 1 Division of Healthcare Quality Promotion, National Center for Emerging and Zoonotic Infectious Diseases, CDC.

Yellow fever vaccine (YF-VAX, Sanofi Pasteur, Swiftwater, Pennsylvania) is a live, attenuated virus vaccine recommended for persons aged ≥9 months who are traveling to or living in areas with risk for yellow fever virus transmission (*1*). For persons of all ages for whom vaccination is indicated, a single subcutaneous injection of 0.5 mL of reconstituted vaccine is used. Because no specific treatment for yellow fever exists, prevention through vaccination is critical to reduce yellow fever–associated morbidity and mortality (*2*). YF-VAX is the only yellow fever vaccine licensed in the United States, and approximately 500,000 doses are distributed annually to vaccinate military and civilian travelers. Yellow fever vaccine is supplied only to designated Yellow Fever Vaccination Centers authorized to issue certificates of yellow fever vaccination. YF-VAX is available in single-dose and 5-dose vials. Single-dose vials of lyophilized (freeze-dried) vaccine are supplied in a package of five vials of vaccine (Figure); five vials of diluent are provided separately (each vial of diluent contains 0.6 mL sodium chloride for injection USP). Five-dose vials are supplied in a package containing one vial (Figure), and diluent is supplied separately in one vial containing 3 mL sodium chloride for injection USP. The manufacturer’s instructions specify that the vaccine is to be used within 60 minutes of reconstituting either the single-dose or the 5-dose vial.

In March 2017, four persons at a single military clinic were vaccinated in error, each receiving an entire 5-dose vial of YF-VAX reconstituted with 0.6 mL of diluent before administration. No specific adverse events were reported; all persons were evaluated in an emergency department (ED) and released. The error was reported to the Vaccine Adverse Event Reporting System (VAERS) (*3*), which prompted CDC to search the VAERS database for similar reports of incorrect dosage administration of YF-VAX. Eleven reports of similar errors in vaccine administration were identified, including a cluster of seven persons vaccinated at another military clinic in 2007 and four other reports (one from a public health clinic in 2010, two from separate military clinics in 2011 and 2013, and one from an unknown type of clinic in 2013). Among the 15 patients identified, five were evaluated in an ED, and one had a doctor’s evaluation in a clinic. Only one report described symptoms; a man aged 30 years was evaluated in an ED for intermittent upper abdominal pain and arm pain 1 day after inadvertent receipt of a 5-dose vial; his symptoms resolved following supportive intravenous treatment. 

Reports of similar administration errors are rare. Three Brazilian reports involved multidose vials of 17-DD yellow fever vaccine (Bio-Manguinhos, Rio de Janeiro, Brazil) used in mass vaccination campaigns (*4*–*6*); 14 health care workers were asymptomatic following receipt of a 25-fold overdose (*4*); one person received a 12.5-fold overdose but was lost to follow up (*5*); and a 45-day clinical follow up of 49 persons who received a 10-fold overdose identified one child who was hospitalized for evaluation of possible acute viscerotropism and recovered (*6*).

Most reports did not involve an adverse event, but the error was costly in terms of follow-up medical evaluation and vaccine waste. Vaccine providers should follow the instructions provided with YF-VAX; preventive measures such as more distinctive packaging and in-service training in clinics that stock both the single and multidose vials might be helpful.

**FIGURE:**
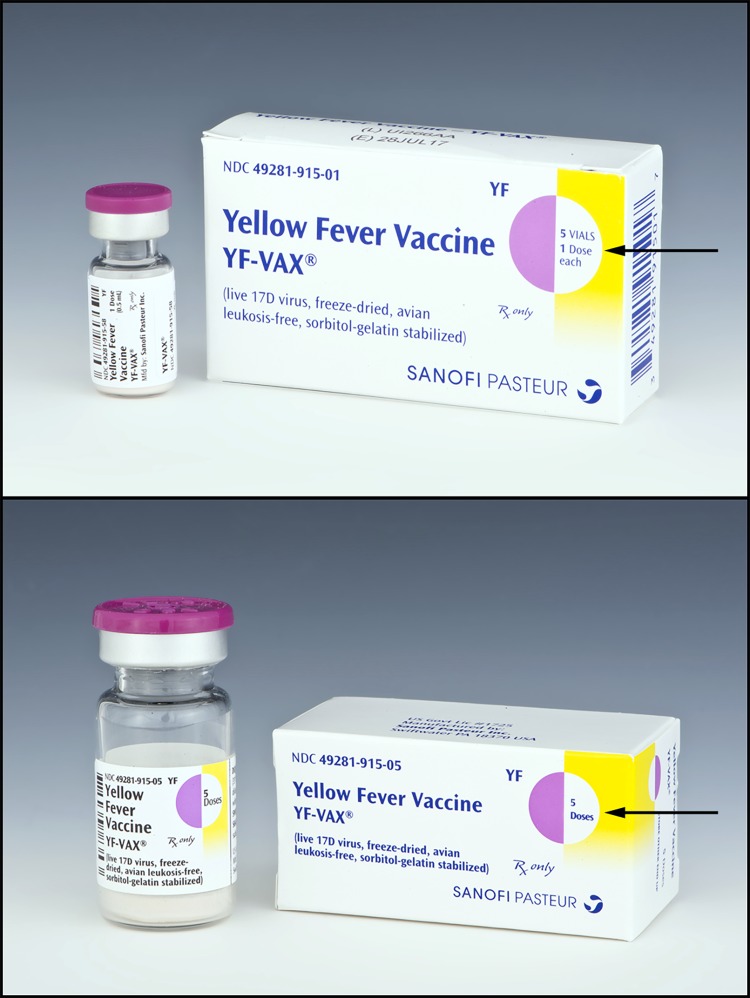
Yellow fever vaccine (YF-VAX, Sanofi Pasteur, Swiftwater, Pennsylvania) supplied as five single-dose vials (top) and one 5-dose vial (bottom)* Photo/Sanofi Pasteur * Arrows indicate package identification of number of doses supplied.
